# Identification of wheel track in the wheat field

**DOI:** 10.1038/s41598-024-51601-x

**Published:** 2024-01-09

**Authors:** Wanhong Zhang

**Affiliations:** Institute of Soil and Water Conservation, Northwest Agriculture & Forestry University, Yangling, 712100 China

**Keywords:** Image processing, Agroecology, Conservation biology

## Abstract

Agriculture machinery navigating along permanent traffic lanes in the farmland may avoid causing extensive soil compaction. However, the permanent traffic lanes are frequently covered up or eliminated by following tillage practices. It is necessary to identify the wheel tracks designed as permanent traffic lanes in order to ensure the agriculture machinery travels along the designated wheel tracks when cultivating the field. This study proposed an identification method of wheel tracks based on the morphological characteristics of wheel tracks and the environmental conditions around the wheel tracks in the wheat fields. The proposed method first utilized the maximum interclass variance to identify the contours of the main part of the wheel track and the shadow regions around the wheel track’s edges. The main part of the wheel tracks was then separated from interference pixels by moving the centerline of the main part of the wheel track, which was derived by skeleton algorithm and curve fitting, towards the right or left edge of the wheel track at a specific distance. In a morphological opening operation, specific linear and circular structural elements were used to segment the shadow regions along the edge of the wheel track. The remaining wheel track was finally recognized by computing the complement of the region identified. After achieving the segmentation of wheel tracks, many reference points near the outside of the wheel track edge in the original image were chosen as fiducial points for evaluating the differences between the actual value and the recognized wheel track edge. The evaluation was based on computing the root mean squared error (RMSE) and the mean absolute error (MAE) of coordinates of reference points and recognized wheel track edge. The results showed that the largest RMSE and MAE were 24.01 pixels (0.0045 m) and 17.32 pixels (0.0032 m), respectively. The low values of RMSE and MAE reveal that the accuracy of the algorithm developed in this study is high, and using this algorithm may segment the wheel track in the wheat field accurately.

## Introduction

The wheel track left in fields by agricultural machinery during operation is known as a field wheel track. These wheel tracks result from the pressure exerted by the wheels of the machines, which compact the soil vertically and cause lateral displacement, resulting in depressions or grooves^1^. The primary factors that contribute to the formation of field wheel tracks are agricultural machinery transporting fertilizers, seeds, tools, and other agricultural materials in the fields; spraying machines, cultivators, seed drills, and harvesters working in fields; and machines rolling the soil to conserve moisture and prevent frost damage in northern wheat fields of China. When the wheel track occurs, the soil porosity gradually decreases, soil bulk density increases, and the soil texture compacts. Changes in soil alter the morphological structure of the roots and impede the roots' ability to absorb deep soil moisture and nutrients. Root and soil changes, in turn, reduce the crops’ ability to withstand natural disasters, limit the increase in crop yields, and hinder water infiltration, exacerbating soil erosion^2–6^.

Soil compaction can be caused by traffic in both the topsoil and subsoil. According to a study conducted by Oomżał and Hodar^7^, it was observed that a depth of 10 cm of topsoil exhibits more susceptibility to soil compaction when subjected to the pressure exerted by agricultural machinery in agriculture. Subsoil compaction in agricultural production has been attributed to increasing the weight and number of passes of agricultural machinery, according to Bakker and Davis^8^. When compaction occurs in the surface layer of soil, human intervention can effectively relieve soil compaction. However, when subsoil compaction occurs, agricultural production will be severely affected, and this negative effect can persist for a long time^5,9^. Several field management strategies have been proposed to reduce soil compaction, particularly subsoil compaction. Braunack et al.^10^ proposed a method to confine field traffic on permanent traffic lanes, in which the permanent traffic lanes and crop beds are distinctly separated. With that method, the average yield of cotton improved by 15% while the area the wheel covered in the lucerne field after each operation decreased from around 70 to around 20%^11,12^. The methods of controlled tracks can potentially lessen the adverse effects of soil compaction on agricultural production^13^. Nevertheless, the agricultural machinery is often unable to precisely travel along the wheel tracks designed as permanent lanes because these tracks are frequently covered up or eliminated by following tillage, which can result in new soil compaction^14,15^. In this sense, the wheel track needs to be recognized and positioned precisely for the tillage method of controlled traffic to be performed effectively.

Currently, the study of wheel tracks mainly concentrates on the forestry and road transport industries. In those studies, most research findings are related to measuring the depth of wheel tracks because the techniques for measuring the depth of wheel tracks are well-established, and changes in soil physical properties and driving safety are closely related to soil depth^16–27^. In the studies, Jia et al.^28^ conducted a study investigating the connection between rut depth and driving safety. The study's findings indicate a rut depth of less than 10 mm is a safe threshold for roads with high driving speeds. Liu et al.^29^ optimized the asphalt mix design using rut depth prediction for asphalt pavement. In their study, Ferenčík et al.^26^ utilized Close-range photogrammetry to quantify the depth of the rut formed by wheeled skidders within the University Forest Enterprise of the Technical University located in central Slovakia. The study by Zheng et al.^30^ examined the impact of rut in asphalt surfaces on the steering stability of autonomous vehicles. The study's results indicate that the critical determinant of the vehicle’s maximum roll angle is the depth of the rut. Compared to the current scientific research document that addresses the depth of wheel tracks, little information has been provided on the recognition of wheel tracks exceeding the circumference of a single tire in the agriculture, forestry, and road transportation industries. However, recognition of the tiny fragment of wheel tracks (area of wheel contact surface) has been reported by Derafshpour^31^. The rate of soil compaction caused by vehicle traffic and the location of the wheel track may be determined easily by recognizing the wheel track, which Ma^32^ has testified in their studies through recognition of the wheel track caused by snow terrain vehicles in the snow. Hence, it is possible to describe the area and location of the wheel tracks in farmland through the recognition of wheel tracks.

Cognition of wheel tracks needs to acquire accurate information about the width, length, and contours because the width, length, and contour are important plane structure features of a wheel track. Currently, two basic methods exist for measuring the geometric dimensions of wheel tracks. One method uses the rulers. The measurement of this method is reliable, but it is time-consuming and laborious and can only allow for a small region at a time^33,34^. Besides, this method cannot describe the wheel track’s contours. Another method uses photogrammetry, a study approach based on photography and image processing. Compared to manual measurement, photogrammetry may capture a broader range of research fields utilizing optical photography equipment^17^ and more accurately reflect the fine details of an object’s shape by applying close-range photography^34,35^. Therefore, photogrammetry is an ideal tool for recognizing the wheel tracks.

Wheat is a widely cultivated crop in the northern regions of China, and moist soil with frequent agricultural machinery traffic during wheat planting and harvesting season often causes distinctive wheel tracks in wheat fields in the Chinese north, which creates favorable conditions for the use of close-up photography to recognize the wheel track in the wheat field. The objective of this study is to present a novel identification technique for wheel tracks in wheat fields. The proposed approach aims to improve the implementation of control traffic strategies in wheat fields.

## Methods

In this study, image processing was based on the Matlab R2020a platform, and the image processing method was demonstrated using the left wheel track of Fig. 2a.

### Images acquisition

**Figure 1:**
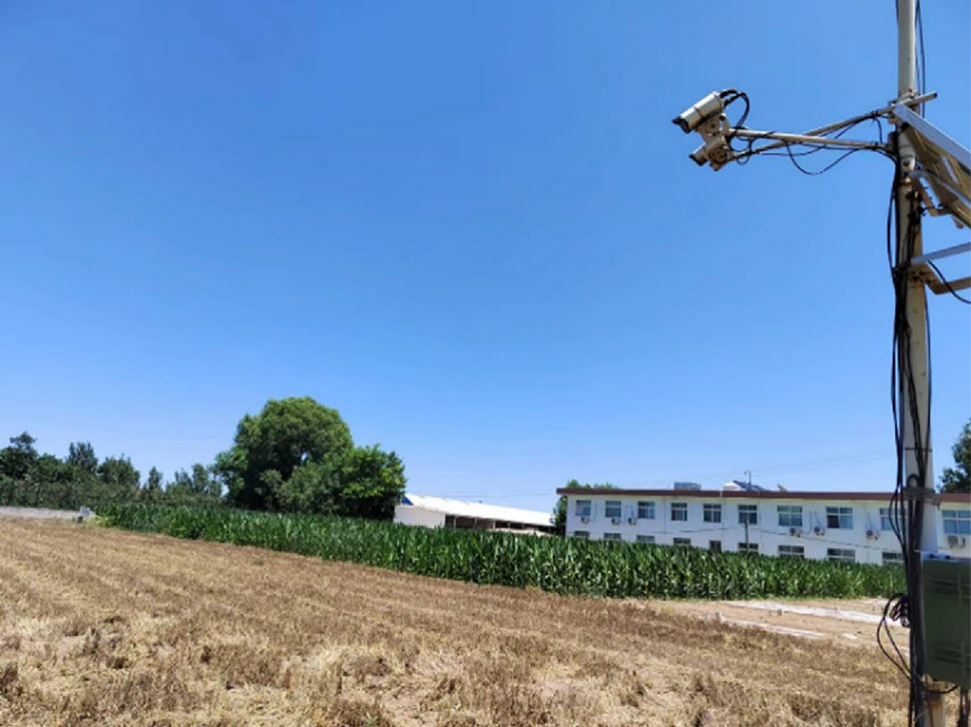
Using the camera to capture the image in the wheat field.

The study was conducted in wheat fields at the National Field Scientific Observation and Research Station of Agricultural Ecosystems in Changwu, Shaanxi province of China. Before starting the experiment, the cameras were installed in the experimental field, with a vertical distance from the ground of about 2.8 m and a lens angle of less than 90° to the horizontal ground (Fig. 1). After finishing the camera settings, the cameras were launched to collect field images. The format of images collected is in a joint photographic expert group (JPG).

### Image filtering

**Figure 2:**
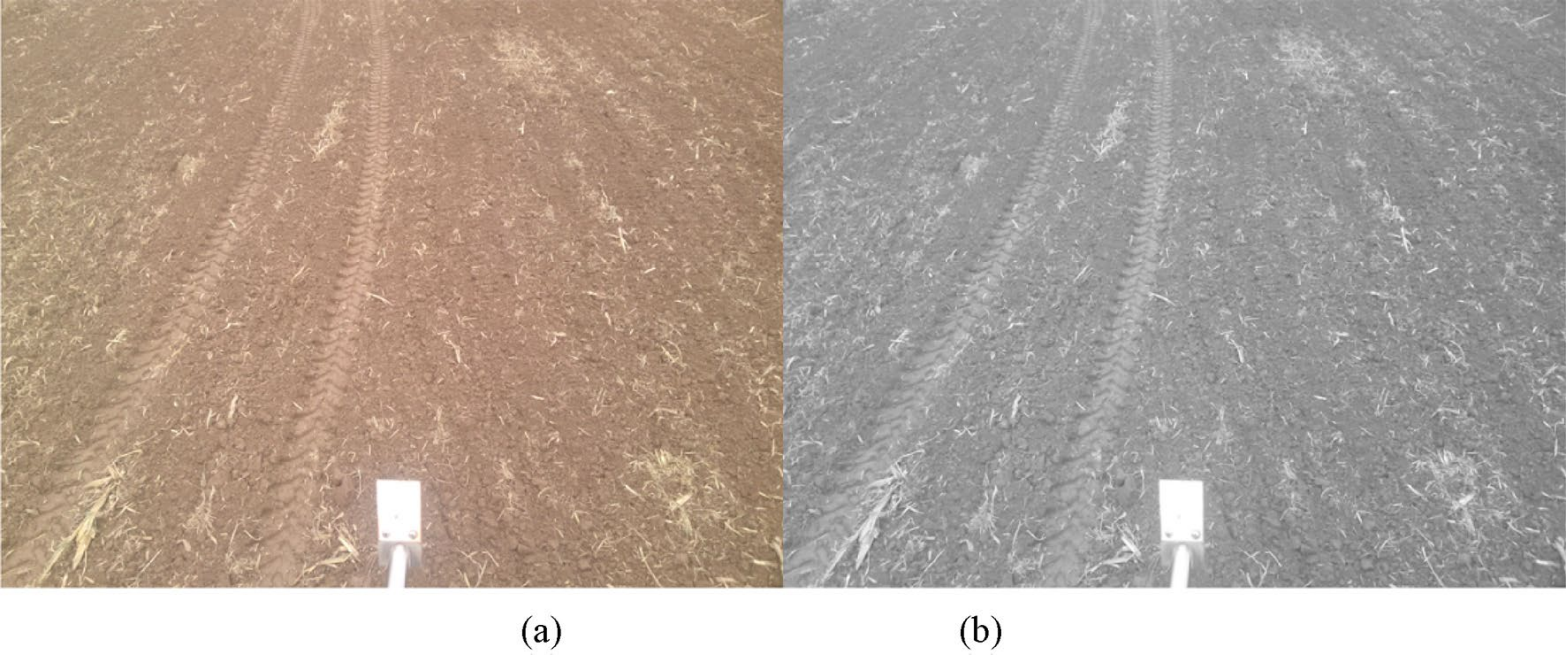
Filtering the image using Gaussian filtering. From left to right: (**a**) original image, (**b**) gray image.

In order to avoid blurring the edges of the background and foreground objects during filtering, Gaussian filtering that maintains the edges was used^36^. This method filters images by adjusting the smoothness levels (the algorithm's default is 650.25 for 8-bit unsigned integer data) and standard deviation values. The regions of the image with more consistent colors are smoothed when the smoothness value is low, while the regions with sharp color variation are smoothed when the smoothness value is high. After establishing the smoothness value, the filtered region can be expanded by raising the Gaussian filter's standard deviation. The study initially converts an RGB image (Fig. 2a) to a grayscale image for the research, followed by filtering. To achieve the highest quality filtering effect, the smoothness values were modified around the default value of smoothness, the different Gaussian filter standard deviations were tried, and the image processing effect was checked visually. The filtered image was displayed in Fig. 2b after the image with the optimal filtering effect was selected and processed.

### Initial image segmentation

**Figure 3:**
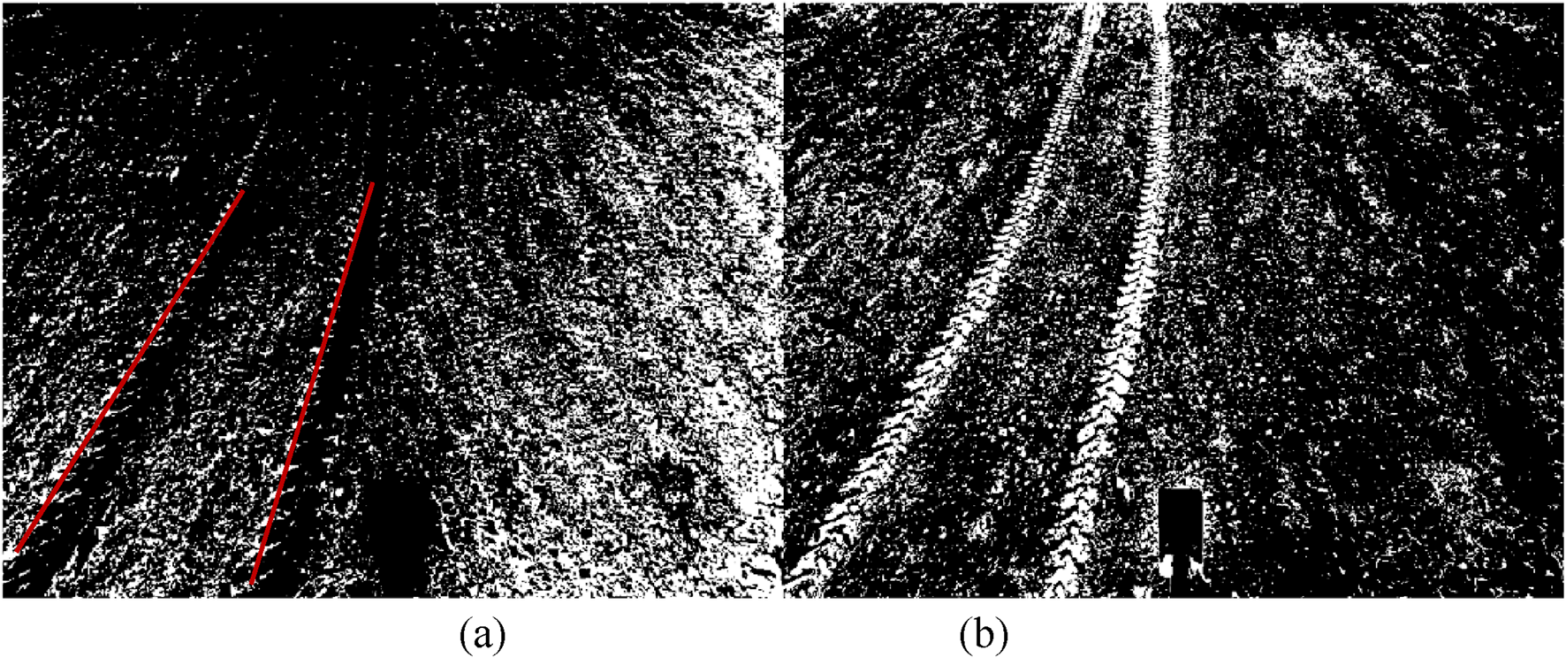
Initial segmentation of the image using the multi-threshold maximum inter-class variance approach. From left to right: (**a**) shadow region of the wheel track, (**b**) main part of the wheel track.

In addition to wheel tracks, surrounding soil, and rectangular white reference objects, it was discovered upon examination of the field images that residual straws and husks from the previous season’s crop were also found within the image (Fig. 2a). The soil and white reference objects surrounding the wheel tracks exhibited distinct color differences from surrounding objects and were thus easily separable by applying thresholding methods. However, the color of the wheel tracks was similar to that of the straw and husk residues in some pots, where the residues were tightly connected to the edges of the wheel tracks. Consequently, it was not easy to distinguish the wheel tracks from the residues using conventional thresholding methods. Nevertheless, the outline and tendency of wheel tracks were discernible. Moreover, the left edge of the wheel tracks featured a distinct shadow region that could be identified to locate the tracks' left boundaries. Thus, this study performed initial image segmentation using the multi-threshold maximum inter-class variance approach (OTSU), followed by image processing techniques to eliminate unwanted components and completely display the wheel tracks' regions. Two binary images were generated as a result of the multi-threshold segmentation. Figure 3a shows the segmented shadow region of the wheel tracks (with a red line indicating the tracks' trend), while Fig. 3b shows the segmented main part of the wheel tracks.

### Recognition of the left edge of the wheel track

**Figure 4:**
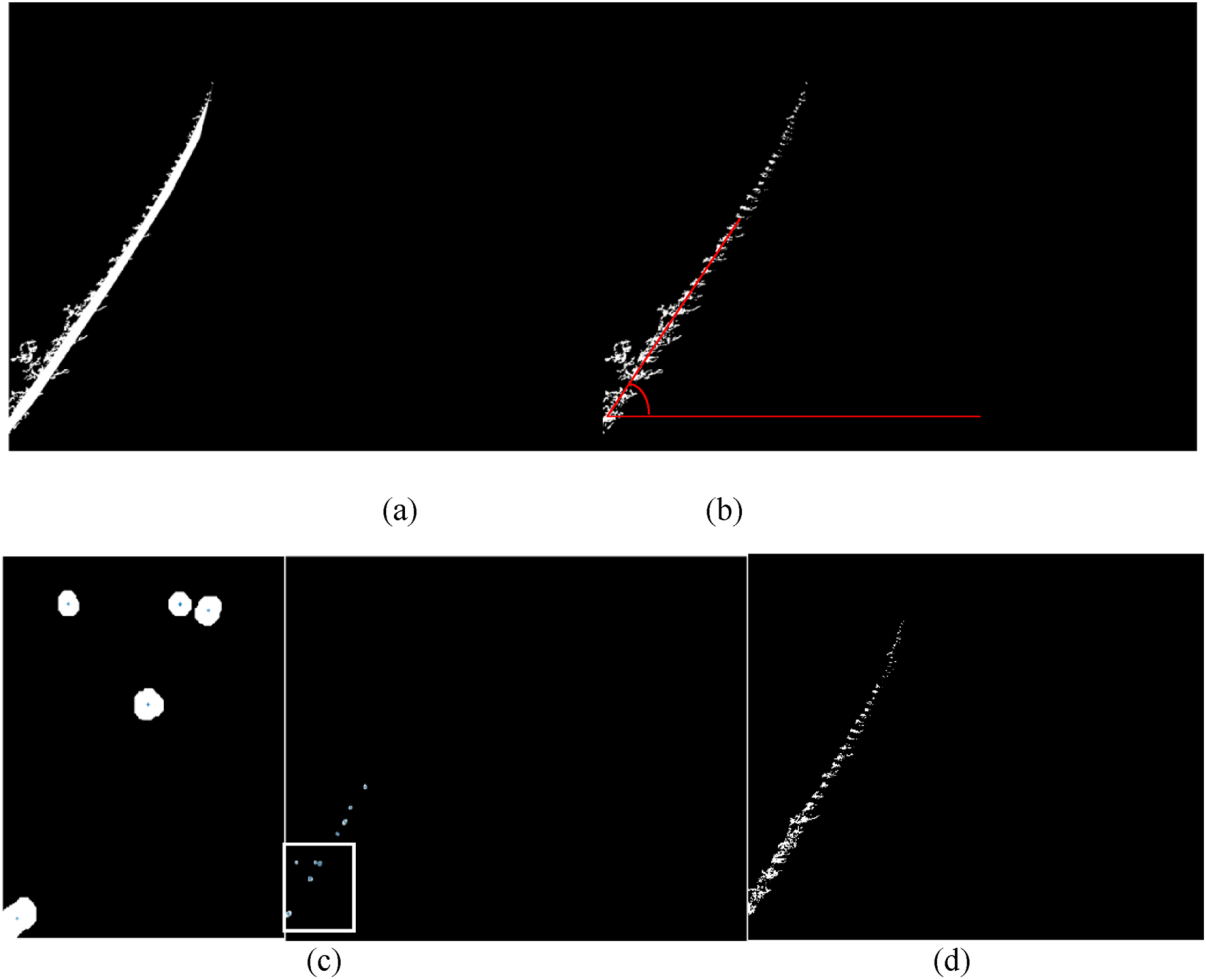
Removing the interference pixels of the edge of the wheel track. (**a**) The connected components of the wheel track, (**b**) removing the connectivity of the wheel track, (**c**) determination of the centroid, (**d**) removing the interference.

Due to the shadow regions of the left edge of the wheel tracks situated at the outermost edge of the wheel tracks, as illustrated in Fig. 3a, this study determines the left edge of the wheel track by identifying the left edge shadow regions. Observation of Fig. 3a revealed that although most of the left edge of the wheel tracks have been presented after the first image segmentation, some region is still tied to surrounding interference pixels, making it difficult to detect the left border of the wheel tracks fully. Observation also showed that the pixels that make up the shadow regions gathered into clusters at the far left of the wheel track, and the clusters were arranged roughly straight and discontinuously. It can also be found from Fig. 3a that the surrounding interference pixels are sparsely distributed near the upper end of the image, relatively far away from the edge of the wheel track in terms of space. The interference pixels are closely arranged around the shadow regions of the left edge of the wheel track at the image’s middle and lower ends. Additionally, in some areas of Fig. 3a, the interference pixels and the shadow region of the edge of the wheel track even combine. Considering the above morphological features of the image, it is feasible to use the morphological opening operation to separate the shadow areas of the left edge of the wheel track from their surrounding interference pixels. In order to effectively separate the shadow regions of the left edge of the wheel tracks, the method of selecting the image area of interest was introduced to connect the discrete pixel clusters of the shadow regions of the left edge of the wheel track to build a connected component. After the connected component was formed, as shown in Fig. 4a, the algorithm for eliminating objects with small areas was employed to clear away the tiny interference pixels around the connected component. Most of the small-size interference pixels adjoining the connected component were then removed by performing the algorithm of clearing away the tiny area objects. The outcome of removing interference pixels of small sizes is shown in Fig. 4b. Figure 4b indicates that some interference pixels are still closely coupled to the shadow regions of the left edge of the wheel tracks, but most interference pixels around the connected component have been removed.

The morphological opening operation was introduced in order to break the connection between the interference pixels and the shadow regions of the left edge of the wheel tracks. Before implementing the morphological opening operation, the structural element style was first chosen since different structural elements will result in varied operation outcomes. Given that the arrangement for the shadow regions of the left edge of wheel tracks is roughly linear, a linear structural element was created. The angle between the trend of the shadow area of the left edge of wheel tracks and the horizontal line (as shown by the red line in Fig. 4b) was employed as a parameter of morphological opening operation to yield the desired results. In this study, the angle between the shadow area of the wheel track edge and the horizontal line was determined using the following algorithm: calculating the diameter of the circle with the same area as each pixel cluster (the shadow regions of the left edge of the wheel tracks were composed of many pixel clusters); creating a histogram of the number of pixel clusters against corresponding diameter values; determining the radius of the circular structural element based on the information from the histogram; designing the circular structural element with the selected diameter value as the parameter and performing a morphological opening operation on pixel clusters in the shadow regions. Following the morphological opening operation, the centroid coordinates of each pixel cluster were established (as shown in Fig. 4c, where the left image is an enlarged vision of the white box). The angle between the trend of the pixel clusters and the horizontal line was achieved based on computing the tangent value between adjacent centroids using the trigonometric function; those values above three times the standard deviation were removed, and the average value of the remaining values was calculated to determine the angle between the trend of pixel clusters and the horizontal line. Choosing the linear structural element and determined angle as parameters to execute the morphological opening operation on the shadow regions of the left edge of the wheel track removed the interference pixels around the shadow area of the left edge of the wheel track, as shown in Fig. 4d.

### Determining the centerline of the main part of the wheel track

**Figure 5:**
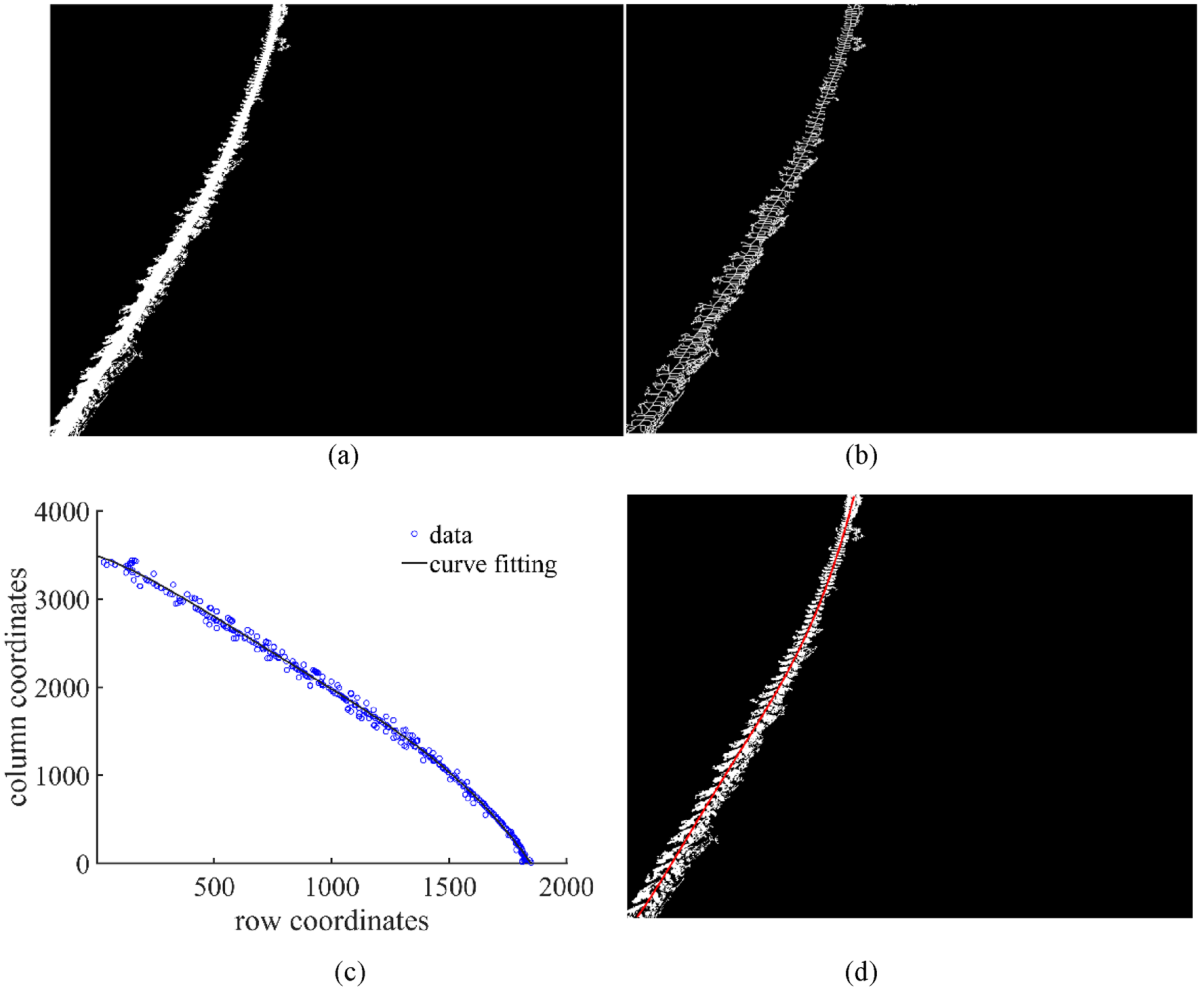
Determination of the centerline of the wheel track based on skeleton algorithm and curve fitting. (**a**) Construction of connected components, (**b**) skeletonization of connected components, (**c**) polynomial fitting based on coordinates of the row and column of midpoints, and (**d**) the centerline of the wheel track, as indicated by the red line.

Figure 3b shows that the main part of the wheel track is approximately symmetrical around the centerline and that the changing trend of the centerline and the edge of the main part of the wheel track have similar patterns. In this study, the centerline of the main part of the wheel tracks can be easily determined by detecting the midpoint. Therefore, separating the wheel track from the interference pixels is viable by moving the centerline a certain distance toward the left or right. A connected component was first constructed in the wheel track’s main part to determine the centerline effectively (Fig. 5a). Then, some interference pixels in the image were removed by area filtering. The skeleton operation on the connected component of the main part of the wheel track was carried out, and resultant skeleton connection points were used to fit the centerline using a fitting function (Fig. 5b). To achieve the best fitting outcomes, polynomial fitting, power function fitting, sine function fitting, and rational function fitting were used separately to fit the skeleton connection points of the main part of the wheel track, and the resultant results were compared. Based on the comparison results, polynomial and rational function fitting has higher fitting goodness and lower root mean square error (Table 1). Nevertheless, polynomial fitting is more in line with the trend of the wheel track’s centerline by observing the fitting curve, as shown in Fig. 5c. Thus, polynomial fitting was used to determine the centerline of the main part of the wheel track. The determined centerline is shown in Fig. 5d.

**Table 1:** Comparison of different curve fittings derived from data on the midpoints of the wheel track.

Function name	Formulas	Goodness of fit	RMSE/pixels
Polynomial	*y* = 0.000 000 014 35*x*^3^−0.000 140 3*x*^2^−0.203 2*x* + 184 4	0.997 1	27.680 4
Power	*y* = − 0.024 26*x*^1.376^ + 183 6	0.997 0	27.825 5
Sine	*y* = 197 5 × sin(0.000 344 4*x* + 1.926)	0.997 0	27.822 4
Rational	*y* = (0.000 043 42*x*^3^−1.241*x*^2^ + 882.1*x* + 104 900 00)/(*x* + 571 2)	0.997 1	27.662 0

### Determining the right and left edges of the main part of the wheel track

**Figure 6:**
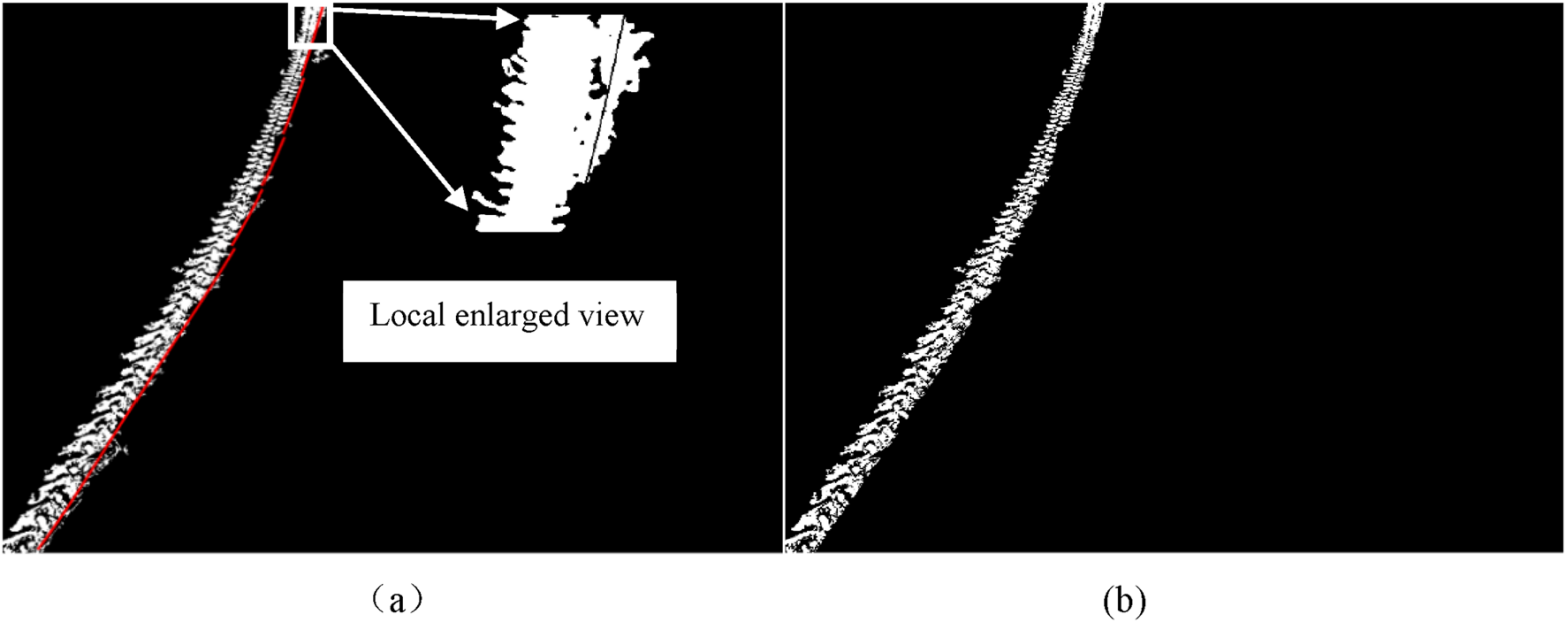
Determination of the right edge of the wheel track based on the movement of the centerline. (**a**) Moving centerline toward the right. (**b**) removing the interference of the main part of the wheel tracks.

After determining the centerline of the main part of the wheel tracks, to remove any interference pixels on the edge of the main part of the wheel tracks and highlight its boundary, the centerline was moved a certain distance toward the left and right to determine the edge of the main part of the wheel track. Observation of Fig. 2a shows that the width of the wheel track gradually decreases with the distance between the wheel track and the camera increase. It could also be observed from Fig. 2a that there is a non-zero angle between the wheel track and the horizontal line due to the curved driving of agricultural machinery in the field. Under the same angle, the trend of the edge of the main part of the wheel track and the centerline all tend toward a straight line. Based on the above image features, the angle between the trend of the main part of the wheel track and the horizontal line is first measured, followed by the measurement of the distance between the left boundary of the main part of the wheel track and the centerline under different angles. The angle between the trend of the wheel track and the horizontal line is determined based on the trigonometric function relationship between them. After determining the angle, the main part of the wheel track was divided into different regions based on the angle. For each region, a target point is manually selected near the left edge of the main part of the wheel track, and the distance between each target point and the centerline in the corresponding region is calculated. According to this distance, the centerline of the corresponding region was moved toward the right or left to clear away interference pixels around the region of the main part of the wheel track (indicated by the red line in Fig. 6a). The wheel track without interference pixels is shown in Fig. 6b.

### Recognition of other areas of the wheel track

**Figure 7:**
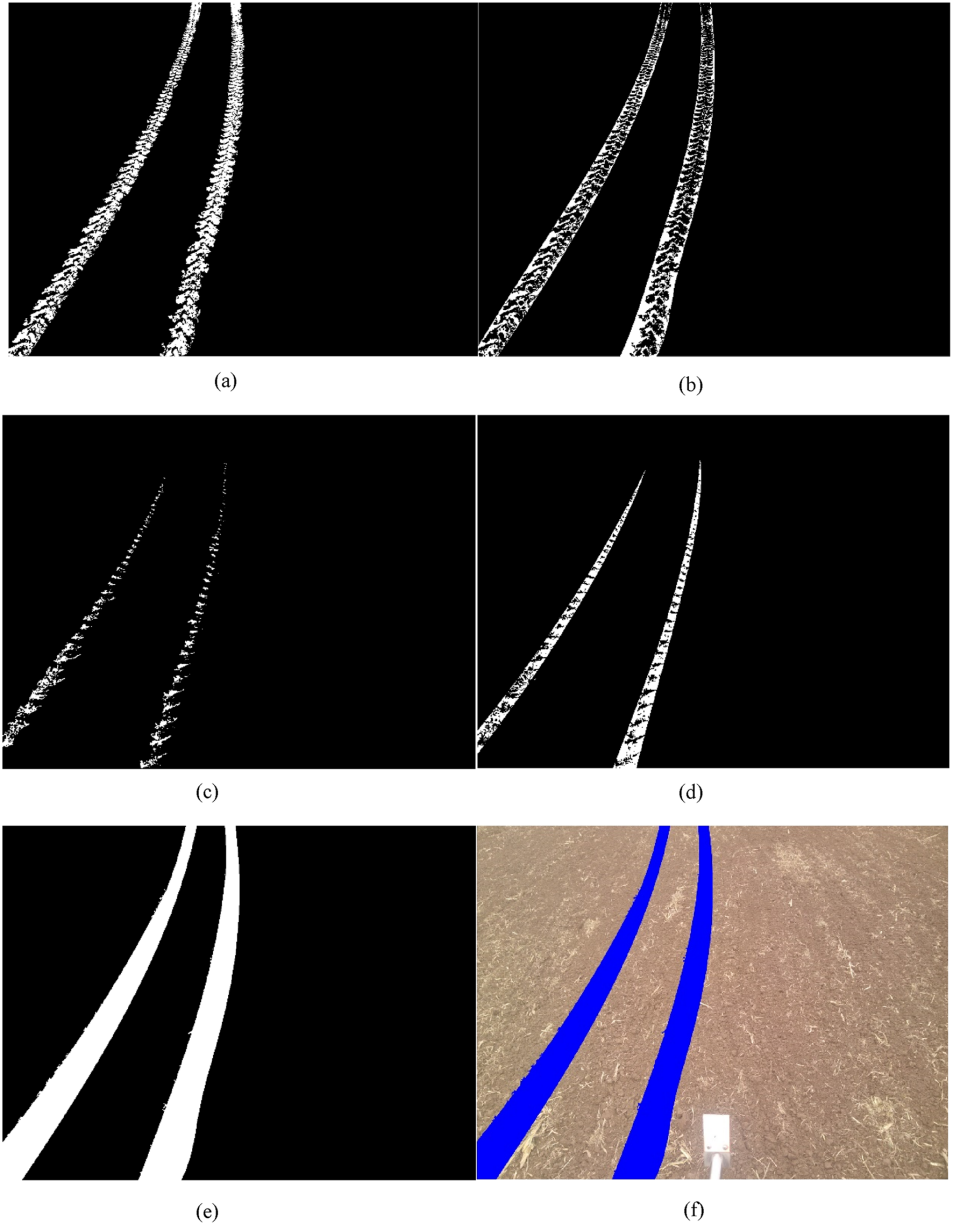
Segmentation of the wheel track in the wheat field. (**a**) The main part of the wheel track, (**b**) the complement of the main part of the wheel track, (**c**) the shadow area of the wheel track, (**d**) the complement of the shadow area of the wheel track, (e) the binary image of the wheel track, (**f**) displaying the wheel track over the original picture.

After applying the Otsu method to segment the wheel track, most of the bright areas caused by the groove of the tires and shadow areas caused by the protrusion of the tires on the left edge of the wheel tracks were identified. Other areas in the wheel tracks were not recognized due to the presence of straw and other factors (Fig. 7a). In order to obtain a complete wheel track, the method of identification of the unrecognized areas in the wheel track was carried out. The study first joined the edge lines of the wheel track in the top, bottom, left, and right directions to form a closed body. Then, the gap inside the closed body was filled using the filling algorithm to form a connected component without holes. In order to show the unrecognized area, the connected component was subtracted from the already identified bright areas in the wheel tracks (Fig. 7b). The recognition method for the bright areas in the shadow area on the left side of the wheel track was the same as described above (Fig. 7c and d).

The method of recognizing the wheel track on the right side of the image was the same as that used for the left side. After recognizing the wheel track on the right side, the tracks on both sides were merged to form a whole wheel track. The recognized wheel track is shown in Fig. 7e and f.

## Results

**Figure 8:**
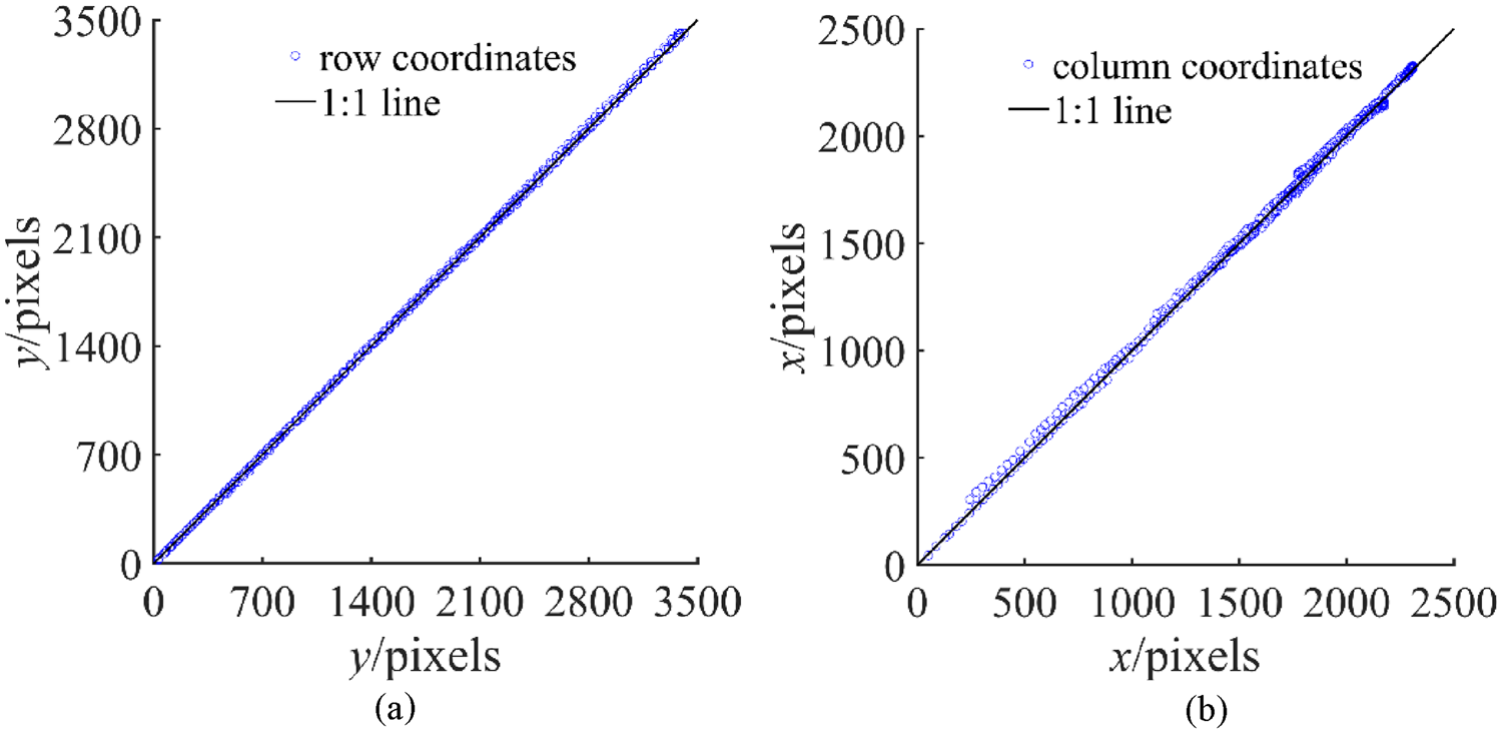
1:1 picture of row and column coordinates. (**a**) Row coordinates, (**b**) column coordinates.

To evaluate segmented results, multiple points were manually selected near the outer edge of the wheel track in the original image, and coordinate information of selected points was extracted. The 1:1 line plot of the reference values against the measurement values was first created based on the coordinate information of the selected points (reference values) and the segmented edge of the wheel track (measured values) (Fig. 8). Then, the root mean squared error (RMSE) and mean absolute error (MAE) values were calculated (Table 2).

**Table 2:** Calculating RMSE and MAE of horizontal and vertical coordinates for the reference values and measured values.

Coordinate	RMSE/pixels	MAE/pixels
Row	12.57	7.35
Column	24.01	17.32

It can be found by observing the 1:1 line plot that the reference values and measure values are closely arranged around the 1:1 line. Compared to the row and the column coordinate plot, the data in the column coordinate plot was more scatted, with a higher RMSE value (24.01 pixels, 0.0045 m) and MAE value (17.32 pixels, 0.0032 m) than those for the row coordinate with an RMSE of 12.57 pixels (0.0023 m) and an MAE of 7.35 pixels (0.0013 m). The relatively small RMSE and MAE values for the row coordinates suggest that the measured values are almost consistent with the reference values. The larger RMSE and MAE values for column coordinates indicate a small gap between the segmented edge of the wheel track and the selected points.

When comparing the calculated outcomes to those of previous research, it was found that Salmivaara et al.^37^ achieved a root RMSE of 0.035 m when employing a Light Detection and Ranging (LiDAR) sensor to measure the depth of wheel tracks. In their investigation of soil surface changes using close-range photogrammetry, Ferencík et al.^26^ found a range of RMSE between 0.026 m and 0.050 m. The RMSE values calculated for this investigation's row and column coordinates were smaller than the reported values.

Based on the completed analysis, it can be inferred that the proximity of the segmented wheel track edge to the ground truth is supported by the close resemblance between the coordinates of the measured edge pixel and those of nearby non-wheel track area points. The low values of the RMSE and MAS further validate this conclusion.

## Discussion

When the foreground and background colors in an image are similar, thresholding methods may lead to under-segmentation or over-segmentation, which may cause errors in numerical calculations based on segmentation results. In order to reduce calculation errors caused by improper image segmentation, the image originating from primary segmentation needs to undergo secondary image processing to correct image segmentation. In this study, some areas of the wheel track edges are similar in color to the background, making it difficult to achieve a complete wheel track using thresholding. However, thresholding can ensure that most wheel track areas are segmented. Therefore, the study uses thresholding for initial segmentation. After the initial segmentation, the contour for the wheel track’s main part and the wheel track’s shadow regions were obtained. Considering the right and left side of the main part of the wheel track is symmetric along the centerline, the trends of the centerline are the same as the edge of the main part of the wheel track, and the study moved the centerline of the wheel track toward edges of the main part of wheel track to segment the main part of wheel track from interference pixels. Before the segmentation of interference pixels adheres to the edge of the main part of the wheel track, it is necessary to determine the centerline of the wheel track first. Currently, there is no way to extract the centerline of the wheel track directly. However, the wheel track’s midpoints are components of its centerline, and the extraction of the midpoints of the wheel track is feasible. So, this study determined the centerline of the wheel track based on determining the midpoints of the wheel track. To ensure the accurate extraction of the midpoints of the wheel track, the study constructed a connected component in the central area of the main part of the wheel track. Then, this study used the skeletonization algorithms to shrink the connected component toward its center to produce many points. The connected component was reduced to many points after the skeletonization. The study used curve fitting methods to fit the scattered points and derived the centerline of the wheel track from the fitting function, which matches the scatted points well. In this study, the method for determining the centerline of the wheel track can provide a reference for other similar studies.

Image distortion often affects the results of quantitative calculation in image processing. The study fully considers the impact of image distortion on determining the wheel track. After identifying the centerline of the main part of the wheel track, the centerline was moved toward the left or right side of the wheel track to determine the edge of the main part of the wheel track. Under the condition that the arrangement of the wheel track trends to a straight line, the new position at which the centerline of the wheel track arrived by moving can be used directly as the left and right edges of the main part of the wheel track. However, when the arrangement of the wheel track is a curve, moving the same distance causes the centerline to deviate from the actual edge line due to the different degrees of image distortion in different directions. In order to solve this problem, the study first calculated the angle between the straight line formed by adjacent points on the centerline of the main part of the wheel track and the horizontal line through trigonometric functions. Then, the study split the whole wheel track into several fragments according to different angles. Finally, moving the curve on each segment of the wheel track locate the corresponding edge of the main part of the wheel track.

The type of structural components utilized for morphological operations affects image processing results. In order to separate the edge of the wheel track from the interference pixels while maintaining the width of the wheel track edge unchanged, this study performed the opening operations with linear structural elements to separate interference pixels from the edge of the wheel track. It is a fact that when the angle of the linear structural element is in line with the trend of the wheel track, the opening operation using the line structural element extends or contracts wheel tracks along the trends of the wheel track. This results in a slight variation in the shadow regions of the wheel track edge in the vertical direction of the line structural element. As a result, the horizontal variation of the wheel track will be greatly reduced. Thus, it is necessary to calculate the angle between the wheel track edge pixels and the horizontal line to achieve better image processing results from the opening operation of the linear structure element. In this study, it is difficult to calculate the angle between the shadow region of the wheel track edge and the horizontal line directly because the shape of the shadow region of the wheel track edge in the binary image is irregular, and their arrangement seems disordered. The circular structural elements may remove smaller objects in the erosion algorithm and make the irregular object regular. In order to detect the changing trend of the shadow region of the wheel track edge, the circular structural element was used to regulate the shape of the shadow region of the wheel track edge. After implementing the morphological operation of circular element on the shadow region of the wheel track edge, the tiny protrusions at the edge of the wheel tracks were removed, the shape of the shadow region of the wheel track edge became regular, and the centroid of each polygon that formed the shadow region of the wheel track tended toward a straight-line arrangement. Since the polygon's centroid tended towards a straight-line arrangement, the angle between the linear structural element and the horizontal line could be readily determined by calculating the angle between the line connecting the polygon centroid and the horizontal line. The mentioned method above can separate the interference pixels attached to the shadow region of the wheel track edge, and this method also provides an example of accurately using structural elements based on target features in morphological operations.

The method proposed in this study was developed using somewhat complex image processing methods and math knowledge. Consequently, it is difficult for individuals responsible for managing farms to apply if they are unfamiliar with image processing and associated mathematics. Moreover, in cases where the wheel track is indistinct, the proposed approach fails to accurately identify it. It is true that the calculation process needs human intervention and cannot be entirely automated, which also is a drawback.

## Conclusions

The multi-threshold algorithm was first used for this study’s initial segmentation of the wheel track in the wheat field. Then, the points around the midpoint of the wheel tracks were identified depending on the skeletonization of the main part of the wheel track. Next, the curve fitting was carried out to derive the centerline of the wheel track based on the identified points. Finally, the wheel track’ edges were separated from the interference pixels by moving the derived centerline toward the left or right side of the main part of the wheel track at a specific distance, and the wheel track in the wheat field was recognized.

The approach mentioned above may also be used to segment wheel tracks in croplands such as corn and soybean fields, among others. Moreover, determining the wheel track location in the wheat field is simple after the methods successfully identify the wheel tracks. In this context, it is possible to conduct continuous studies on the same wheel tracks within a wheat field for multiple years, even if the wheel tracks become indistinct or imperceptible due to crop coverage and machinery cultivating. Nevertheless, the current automation procedure of the algorithm presented in this study needs to be revised. Therefore, the next step will increase the automated operation of the method.

## Data Availability

Data used in the study is available from the authors on a reasonable request.
